# Supplementary material for: Putative Biomarkers for Acute Pulmonary Embolism in Exhaled Breath Condensate
Source: J Clin Med. 2021 Nov 4;10(21):5165. doi: 10.3390/jcm10215165 (PMC8584843; doi:10.3390/jcm10215165)
Supplement: Supplementary file 1 [file jcm-10-05165-s001.zip › jcm-1405627-supplementary.pdf]

Supplementals

Supplemental tables

Table S1. Discovery-based differential expression analysis based on 827 proteins in EBC collected after PE compared with before PE or negative controls.

Majority protein IDs	Protein name	Gene name	Early Post PE vs. Pre PE		Late Post PE vs. Pre PE		Early Post PE vs Early Post C		Late Post PE vs. Late Post C		Early Post C vs.Pre C		Late Post C vs. Pre C	
**Positive fold changes (ie higher amount after PE)**			Fold change	p-value	Fold change	p-value	Fold change	p-value	Fold change	p-value	Fold change	p-value	Fold change	p-value
Q9Y6R7	IgGFc-binding protein	FCGBP	17.1	0.09										
P10909	Clusterin	CLU	12.3	0.06										
P01833	Polymeric immunoglobulin receptor	PIGR	10.6	0.09										
P05090	Apolipoprotein D	APOD	7.6	<0.01										
P09571	Serotransferrin	TF	6.7	0.06										
P50828	Hemopexin	HPX	6.3	0.04										
P68871	Hemoglobin subunit beta	HBB*	6.3	0.05										
P08835	Serum albumin	ALB	5.6	0.10	14.5	0.02								
P04745	Alpha-amylase 1	AMY1A	4.6	<0.01	2.2	0.10								
P02751	Fibronectin	FN1	4.4	0.09										
P61626	Lysozyme C	LYZ	3.5	0.05	4.5	0.04							0.1	0.07
P25788	Proteasome subunit alpha type-3	PSMA3	3.2	0.08										
Q9HC84	Mucin-5B	MUC5B	3.2	0.06										
P31025	Lipocalin-1	LCN1	2.6	0.04										
Q13867	Bleomycin hydrolase	BLMH	1.9	0.02	1.7	0.03	2.6	0.07						
O43548	Protein-glutamine gamma-glutamyltransferase 5	TGM5	1.8	0.07										
Q9GZP4	PITH domain-containing protein 1	PITHD1	1.8	0.05										
P01040	Cystatin-A	CSTA	1.7	0.09			2.0	0.10						
Q96P63	Serpin B12	SERPINB12	1.7	0.04			2.3	0.01						
Q8IW75	Serpin A12	SERPINA12	1.6	0.10										
Q02413	Desmoglein-1	DSG1	1.5	0.04			2.0	0.06						
P35606	Coatomer subunit beta	COPB2	1.4	0.10										
P18648	Apolipoprotein A-I	APOA1			9.0	0.04								
P02788	Lactotransferrin	LTF			8.2	0.04								
P02067	Hemoglobin subunit beta	HBB*			4.0	0.10								
O60784	Target of Myb protein 1	TOM1			2.5	0.02								
Q007T2	Cell division control protein 42 homolog	CDC42			2.5	0.04								

Q9UJ70	N-acetyl-D-glucosamine kinase	NAGK			2.4	0.02								
P11413	Glucose-6-phosphate 1-dehydrogenase	G6PD			2.0	0.05								
P30519	Heme oxygenase 2	HMOX2			2.0	0.05								
Q6QAQ1	Actin, cytoplasmic 1	ACTB			1.9	0.02								
P55263	Adenosine kinase	ADK			1.9	0.02								
P12236	ADP/ATP translocase 3	SLC25A6			1.8	0.08								
P26234	Vinculin	VCL			1.5	0.04								
P61978	Heterogeneous nuclear ribonucleoprotein K	HNRNPK			1.5	0.08								
O60664	Perilipin-3	PLIN3			1.4	0.04								
Q14574	Desmocollin-3	DSC3			1.4	0.06								
P07384	Calpain-1 catalytic subunit	CAPN1			1.4	0.09								
Q07960	Rho GTPase-activating protein 1	ARHGAP1			1.3	0.05								
P25311	Zinc-alpha-2-glycoprotein	AZGP1					3.9	0.03						
P22735	Protein-glutamine gamma-glutamyltransferase K	TGM1					2.6	0.04						
P13473-2	Lysosome-associated membrane glycoprotein 2	LAMP2					2.2	0.08						
P26641	Elongation factor 1-gamma	EEF1G					2.2	0.08						
Q06830	Peroxiredoxin-1	PRDX1					2.2	0.03						
P35908	Keratin, type II cytoskeletal 2 epidermal	KRT2					2.2	0.06						
O75223	Gamma-glutamylcyclotransferase	GGCT					2.1	0.09			0.4	0.09		
P09525	Annexin A4	ANXA4					1.8	0.03						
Q15517	Corneodesmosin	CDSN					1.6	0.04						
P01965	Hemoglobin subunit alpha	HBA							22.7	0.07				
P62851	40S ribosomal protein S25	RPS25							1.6	0.09				
Q16719	Kynureninase	KYNU							1.4	0.04				
**Negative fold changes (ie lower amount after PE)**														
Q29205	60S ribosomal protein L11	RPL11	0.2	0.04										
P11940	Polyadenylate-binding protein 1	PABPC1	0.2	<0.01			0.3	0.01						
P55072	Transitional endoplasmic reticulum ATPase	VCP	0.2	<0.01			0.2	0.04						
P19971	Thymidine phosphorylase	TYMP	0.2	0.02					0.3	0.10			12.3	0.08
Q8IYT4	Katanin p60 ATPase-containing subunit A-like 2	KATNAL2	0.3	0.04	0.3	0.08								
Q2XVP4	Tubulin alpha-1B chain	TUBA1B	0.3	<0.01			0.1	<0.01			9.1	0.04		
A8K2U0	Alpha-2-macroglobulin-like protein 1	A2ML1	0.3	<0.01			0.2	0.01			6.6	0.02	6.5	0.08
P58107	Epiplakin	EPPK1	0.3	0.02										
P80188	Neutrophil gelatinase-associated lipocalin	LCN2	0.3	<0.01	0.2	<0.01			0.3	0.02				
P48594	Serpin B4	SERPINB4	0.3	<0.01	0.4	0.04								
O43313	ATM interactor	ATMIN	0.3	0.05	0.4	0.09								
P14324	Farnesyl pyrophosphate synthase	FDPS	0.3	0.01										
P04075	Fructose-bisphosphate aldolase A	ALDOA	0.3	0.01	0.5	0.07	0.1	<0.01			4.8	0.07		

P68371	Tubulin beta-4B chain	TUBB4B	0.3	0.03			0.2	0.01						
P35579	Myosin-9	MYH9	0.3	0.04										
Q5VTE0	Putative elongation factor 1-alpha-like 3	EEF1A1P5	0.3	0.02			0.2	<0.01			7.0	0.08		
P23284	Peptidyl-prolyl cis-trans isomerase B	PPIB	0.3	0.02	0.4	0.03	0.5	0.05						
P50990	T-complex protein 1 subunit theta	CCT8	0.4	0.10										
O43707	Alpha-actinin-4	ACTN4	0.4	0.04			0.3	0.05						
P13639	Elongation factor 2	EEF2	0.4	0.10										
P62750	60S ribosomal protein L23a	RPL23A	0.4	0.04										
P01857	Ig gamma-1 chain C region	IGHG1	0.4	0.06	0.2	0.01			0.3	0.06				
P47929	Galectin-7	LGALS7	0.4	0.04									2.9	0.05
P31947	14-3-3 protein sigma	SFN	0.4	0.01			0.3	0.07					3.8	0.05
P17900	Ganglioside GM2 activator	GM2A	0.4	0.08										
P06733	Alpha-enolase	ENO1	0.4	0.02									3.4	0.02
Q13835	Plakophilin-1	PKP1	0.4	0.05										
P09211	Glutathione S-transferase P	GSTP1	0.4	0.01									6.0	0.03
P60174	Triosephosphate isomerase	TPI1	0.5	0.01	0.5	0.07								
O60218	Aldo-keto reductase family 1 member B10	AKR1B10	0.5	0.10			0.4	<0.01						
P01024	Complement C3	C3	0.5	0.04	0.3	0.05	0.4	0.08			4.0	0.08		
P63261	Actin, cytoplasmic 2	ACTG1	0.5	0.01			0.3	0.01			3.3	0.07	2.5	0.08
P00558	Phosphoglycerate kinase 1	PGK1	0.5	0.05										
P36952	Serpin B5	SERPINB5	0.5	0.04										
P29508	Serpin B3	SERPINB3	0.5	0.05										
P15924	Desmoplakin	DSP	0.5	0.05										
P29401	Transketolase	TKT	0.5	0.08			0.5	0.08						
O02705	Heat shock protein HSP 90-alpha	HSP90AA1	0.5	0.06			0.3	0.02			14.9	0.03	7.4	0.05
P05387	60S acidic ribosomal protein P2	RPLP2	0.5	0.04	0.5	0.02								
P27482	Calmodulin-like protein 3	CALML3	0.5	0.06	0.5	0.03								
P10668	Cofilin-1	CFL1	0.6	0.03										
Q9NZT1	Calmodulin-like protein 5	CALML5	0.6	0.04	0.5	0.01								
P04792	Heat shock protein beta-1	HSPB1	0.6	0.05										
P52565	Rho GDP-dissociation inhibitor 1	ARHGDIA	0.6	0.03			0.5	0.03						
P26599	Polypyrimidine tract-binding protein 1	PTBP1	0.7	0.05			0.6	0.05						
P11021	78 kDa glucose-regulated protein	HSPA5	0.7	0.07			0.6	0.07						
P04062	Glucosylceramidase	GBA	0.7	0.08										
Q15008	26S proteasome non-ATPase regulatory subunit 6	PSMD6			0.2	<0.01								
P61604	10 kDa heat shock protein, mitochondrial	HSPE1			0.2	0.10								
P29700	Alpha-2-HS-glycoprotein	AHSG			0.3	0.10								
Q19S50	Signal transducer and activator of transcription 3	STAT3			0.3	0.10								

P29373	Cellular retinoic acid-binding protein 2	CRABP2			0.4	0.05								
P01859	Ig gamma-2 chain C region	IGHG2			0.4	0.07			0.4	0.07				
P79263	Inter-alpha-trypsin inhibitor heavy chain H4	ITIH4			0.4	0.09								
P62318	Small nuclear ribonucleoprotein Sm D3	SNRPD3			0.4	<0.01								
P0CG06	Ig lambda-3 chain C regions	IGLC3			0.4	0.04								
P01834	Ig kappa chain C region	IGKC			0.5	0.10			0.3	0.07				
Q9NZH8	Interleukin-36 gamma	IL36G			0.6	0.04			0.5	0.08				
P40305	Interferon alpha-inducible protein 27, mitochondrial	IFI27			0.6	0.10								
P13797	Plastin-3	PLS3			0.6	0.05								
Q08211	ATP-dependent RNA helicase A	DHX9			0.6	0.04								
O95819	Mitogen-activated protein kinase kinase kinase kinase 4	MAP4K4			0.6	0.09								
P40925	Malate dehydrogenase, cytoplasmic	MDH1			0.7	0.03			0.5	0.03				
P30040	Endoplasmic reticulum resident protein 29	ERP29			0.7	0.09			0.7	0.09				
P25789	Proteasome subunit alpha type-4	PSMA4			0.7	0.04								
P63053	Ubiquitin-60S ribosomal protein L40	UBA52			0.7	0.10								
Q9UL46	Proteasome activator complex subunit 2	PSME2			0.8	0.01								
P10412	Histone H1.4	HIST1H1E					0.2	0.02						
P20305	Gelsolin	GSN					0.2	0.08						
P06576	ATP synthase subunit beta, mitochondrial	ATP5B					0.2	0.05						
P62081	40S ribosomal protein S7	RPS7					0.2	0.02						
Q16777	Histone H2A type 2-C	HIST2H2AC					0.3	0.08			8.3	0.06	9.2	0.05
P16401	Histone H1.5	HIST1H1B					0.3	0.07						
P07237	Protein disulfide-isomerase	P4HB					0.3	0.06						
Q8WVV4	Protein POF1B	POF1B					0.3	0.01						
P67985	60S ribosomal protein L22	RPL22					0.3	0.09						
P52907	F-actin-capping protein subunit alpha-1	CAPZA1					0.4	0.01						
P62701	40S ribosomal protein S4, X isoform	RPS4X					0.4	0.06						
P62937	Peptidyl-prolyl cis-trans isomerase A	PPIA					0.4	0.02			3.4	0.03		
P15311	Ezrin	EZR					0.4	0.01						
Q9Y446	Plakophilin-3	PKP3					0.4	0.05						
P63104	14-3-3 protein zeta/delta	YWHAZ					0.4	0.03			2.4	0.02		
P84085	ADP-ribosylation factor 5	ARF5					0.4	0.10						
Q6ZNF0	Iron/zinc purple acid phosphatase-like protein	PAPL					0.4	0.05						
P50395	Rab GDP dissociation inhibitor beta	GDI2					0.5	0.08						
P06748	Nucleophosmin	NPM1					0.5	0.09						
Q13765	Nascent polypeptide-associated complex subunit alpha	NACA					0.5	0.08						

P51149	Ras-related protein Rab-7a	RAB7A					0.6	0.04						
Q86V81	THO complex subunit 4	ALYREF					0.7	0.09						
P31949	Protein S100-A11	S100A11							0.4	0.05				
P11708	Malate dehydrogenase, cytoplasmic	MDH1							0.5	0.03				
P38606	V-type proton ATPase catalytic subunit A	ATP6V1A							0.5	0.09				
P30101	Protein disulfide-isomerase A3	PDIA3							0.5	0.07				
P07339	Cathepsin D	CTSD							0.6	0.08				
P60900	Proteasome subunit alpha type-6	PSMA6							0.6	0.05				
*Two variants of HBB														

Table S2. Protein concentrations in the EBC.

	PE animals			Negative controls			
	**Mean ± SEM**	**95 %CI**	**ANOVA p-value**	**Mean ± SEM**	**95 % CI**	**ANOVA p-value**	**p-value**
**Pre (baseline)** (µg/ml)	2.81 ± 0.13	2.54 to 3.08		3.05 ± 0.18	2.49 to 3.68		0.35
**Early Post*** (µg/ml)	2.81 ± 0.11	2.56 to 3.06		3.03 ± 0.28	1.81 to 4.26		0.43
**Late Post** (µg/ml)	2.89 ± 0.18	2.51 to 3.28		3.40 ± 0.52	1.73 to 5.06		0.26
			0.89			0.50	
*Extreme outlier in pig number 7 Post PE excluded							

Table S3. Proteins in the condensate from the mechanical ventilator compared with the Pre PE and Pre C samples (N=19)
For proteins only present in one of the 19 Pre PE samples a p-value was not calculated (NA)

Majority protein IDs	Protein names	Gene names	Fold change Pre samples/MV	p-value
P06576	ATP synthase subunit beta, mitochondrial	ATP5B	15.8	<0.01
P62802	Histone H4	HIST1H4A	13.0	<0.01
P09571			12.9	<0.01
Q8TDL5	BPI fold-containing family B member 1	BPIFB1	12.7	NA
Q2XVP4	Tubulin alpha-1B chain	TUBA1B	10.7	<0.01
P01846			7.8	0.02
P40227	T-complex protein 1 subunit zeta	CCT6A	7.6	<0.01
P62081	40S ribosomal protein S7	RPS7	7.3	<0.01
P01965			6.6	0.06
O00299	Chloride intracellular channel protein 1	CLIC1	6.5	<0.01
Q09666	Neuroblast differentiation-associated protein AHNAK	AHNAK	6.3	<0.01
Q86XP0	Cytosolic phospholipase A2 delta	PLA2G4D	6.1	0.02
P50447			6.0	0.03
Q16777	Histone H2A type 2-C	HIST2H2AC	5.8	<0.01
P15311	Ezrin	EZR	5.7	<0.01
P0DMV9	Heat shock 70 kDa protein 1B	HSPA1B	5.5	<0.01
P46405	40S ribosomal protein S12	RPS12	5.5	<0.01
P06748-3	Nucleophosmin	NPM1	5.4	<0.01
Q9UN36-3	Protein NDRG2	NDRG2	5.1	<0.01
P16403	Histone H1.2	HIST1H1C	4.7	0.03
P46940	Ras GTPase-activating-like protein IQGAP1	IQGAP1	4.5	0.01
P62937	Peptidyl-prolyl cis-trans isomerase A	PPIA	4.5	<0.01
P49327	Fatty acid synthase	FASN	4.5	0.07
P49368	T-complex protein 1 subunit gamma	CCT3	4.2	<0.01
P50991	T-complex protein 1 subunit delta	CCT4	4.2	0.03
Q99879	Histone H2B type 1-M	HIST1H2BM	4.1	<0.01
P37802	Transgelin-2	TAGLN2	3.9	<0.01
P16401	Histone H1.5	HIST1H1B	3.9	<0.01
P30050	60S ribosomal protein L12	RPL12	3.9	<0.01
P35579	Myosin-9	MYH9	3.9	<0.01
P14632			3.7	0.12
P07476	Involucrin	IVL	3.6	0.05
P05091-2	Aldehyde dehydrogenase, mitochondrial	ALDH2	3.4	<0.01
Q9H0P0-3	Cytosolic 5-nucleotidase 3A	NT5C3A	3.4	0.02
Q07065	Cytoskeleton-associated protein 4	CKAP4	3.3	0.01
Q13835-2	Plakophilin-1	PKP1	3.3	<0.01
P52597	Heterogeneous nuclear ribonucleoprotein F	HNRNPF	3.0	<0.01
Q15828	Cystatin-M	CST6	3.0	0.06
P62333	26S protease regulatory subunit 10B	PSMC6	3.0	0.01
Q08380	Galectin-3-binding protein	LGALS3BP	3.0	0.01
P14174	Macrophage migration inhibitory factor	MIF	2.9	<0.01
Q5VTE0	Putative elongation factor 1-alpha-like 3	EEF1A1P5	2.9	0.01
O75369-2	Filamin-B	FLNB	2.7	0.01
P61160	Actin-related protein 2	ACTR2	2.7	0.02
P21281	V-type proton ATPase subunit B, brain isoform	ATP6V1B2	2.7	<0.01
Q9NYU2-2	UDP-glucose:glycoprotein glucosyltransferase 1	UGGT1	2.7	0.32
P67985	60S ribosomal protein L22	RPL22	2.7	0.01
P68371	Tubulin beta-4B chain	TUBB4B	2.7	0.04
P29401	Transketolase	TKT	2.6	<0.01
Q9ULZ3-3	Apoptosis-associated speck-like protein containing a CARD	PYCARD	2.6	0.25
P08238	Heat shock protein HSP 90-beta	HSP90AB1	2.6	<0.01
P51149	Ras-related protein Rab-7a	RAB7A	2.5	<0.01
P61978-3	Heterogeneous nuclear ribonucleoprotein K	HNRNPK	2.5	<0.01

P12532	Creatine kinase U-type, mitochondrial	CKMT1A	2.5	0.05
P35321	Cornifin-A	SPRR1A	2.5	0.21
Q6NUJ1	Proactivator polypeptide-like 1	PSAPL1	2.5	<0.01
P07108	Acyl-CoA-binding protein	DBI	2.5	<0.01
P32926	Desmoglein-3	DSG3	2.5	<0.01
Q52NJ1	Ras-related protein Rab-11A	RAB11A	2.4	0.02
P06753-5	Tropomyosin alpha-3 chain	TPM3	2.4	<0.01
P26641	Elongation factor 1-gamma	EEF1G	2.4	0.03
P63221	40S ribosomal protein S21	RPS21	2.3	0.01
O15145	Actin-related protein 2/3 complex subunit 3	ARPC3	2.3	0.06
Q9UBH0	Interleukin-36 receptor antagonist protein	IL36RN	2.3	0.12
P30101	Protein disulfide-isomerase A3	PDIA3	2.2	0.05
P01009-2	Alpha-1-antitrypsin	SERPINA1	2.2	0.03
P01859	Ig gamma-2 chain C region	IGHG2	2.2	0.01
O15173	Membrane-associated progesterone receptor component 2	PGRMC2	2.2	0.08
Q96QA5	Gasdermin-A	GSDMA	2.2	<0.01
P04083	Annexin A1	ANXA1	2.2	0.05
Q14204	Cytoplasmic dynein 1 heavy chain 1	DYNC1H1	2.2	0.06
P50990-3	T-complex protein 1 subunit theta	CCT8	2.2	0.01
O00204-2	Sulfotransferase family cytosolic 2B member 1	SULT2B1	2.1	0.16
P10668	Cofilin-1	CFL1	2.1	<0.01
P68366-2	Tubulin alpha-4A chain	TUBA4A	2.1	0.04
P40199	Carcinoembryonic antigen-related cell adhesion molecule 6	CEACAM6	2.1	0.57
P40121	Macrophage-capping protein	CAPG	2.1	<0.01
Q8NEX9	Short-chain dehydrogenase/reductase family 9C member 7	SDR9C7	2.1	0.01
P80021	ATP synthase subunit alpha, mitochondrial	ATP5A1	2.1	0.20
O43707	Alpha-actinin-4	ACTN4	2.1	0.02
Q19PY3	tRNA-splicing ligase RtcB homolog	RTCB	2.1	0.01
Q9BQ50-2	Three prime repair exonuclease 2	TREX2	2.0	0.03
P80188	Neutrophil gelatinase-associated lipocalin	LCN2	2.0	0.01
P01834	Ig kappa chain C region	IGKC	2.0	0.02
P61158	Actin-related protein 3	ACTR3	2.0	0.03
O95833	Chloride intracellular channel protein 3	CLIC3	2.0	0.06
Q99536	Synaptic vesicle membrane protein VAT-1 homolog	VAT1	2.0	0.08
P23284	Peptidyl-prolyl cis-trans isomerase B	PPIB	2.0	0.04
P01857	Ig gamma-1 chain C region	IGHG1	2.0	0.07
Q9BW30	Tubulin polymerization-promoting protein family member 3	TPPP3	2.0	<0.01
Q8TE68-2	Epidermal growth factor receptor kinase substrate 8-like protein 1	EPS8L1	1.9	0.05
P27797	Calreticulin	CALR	1.9	<0.01
P29373	Cellular retinoic acid-binding protein 2	CRABP2	1.9	0.05
P13489	Ribonuclease inhibitor	RNH1	1.9	0.13
Q9Y5Z4	Heme-binding protein 2	HEBP2	1.8	0.17
Q7KZF4	Staphylococcal nuclease domain-containing protein 1	SND1	1.8	0.21
P0CG06	Ig lambda-3 chain C regions	IGLC3	1.8	0.07
P12081-3	Histidine--tRNA ligase, cytoplasmic	HARS	1.8	0.02
P05120	Plasminogen activator inhibitor 2	SERPINB2	1.8	0.02
P01619	Ig kappa chain V-III region B6	IGKV3D-20	1.8	0.01
P58107	Epiplakin	EPPK1	1.7	0.15
Q96MZ4	Protein FAM218A	FAM218A	1.7	0.04
P11142	Heat shock cognate 71 kDa protein	HSPA8	1.7	0.05
Q6ZNF0	Iron/zinc purple acid phosphatase-like protein	PAPL	1.7	0.11
P06744	Glucose-6-phosphate isomerase	GPI	1.7	0.16
P62263	40S ribosomal protein S14	RPS14	1.7	<0.01
O02705			1.7	0.11
P25786	Proteasome subunit alpha type-1	PSMA1	1.7	0.07
Q06830	Peroxiredoxin-1	PRDX1	1.7	<0.01
P48594	Serpin B4	SERPINB4	1.7	0.10
Q8N5F7	NF-kappa-B-activating protein	NKAP	1.6	0.20
P63104	14-3-3 protein zeta/delta	YWHAZ	1.6	0.06

P84085	ADP-ribosylation factor 5	ARF5	1.6	0.20
P08670	Vimentin	VIM	1.6	0.03
P07355	Annexin A2	ANXA2	1.6	0.02
P20305-2			1.6	0.06
P09211	Glutathione S-transferase P	GSTP1	1.6	0.08
P49720	Proteasome subunit beta type-3	PSMB3	1.6	0.03
Q9NZH8	Interleukin-36 gamma	IL36G	1.5	0.01
P08758	Annexin A5	ANXA5	1.5	0.10
P11021	78 kDa glucose-regulated protein	HSPA5	1.5	0.01
O60218	Aldo-keto reductase family 1 member B10	AKR1B10	1.5	0.08
P37837	Transaldolase	TALDO1	1.5	0.02
P08311	Cathepsin G	CTSG	1.5	NA
P63053	Ubiquitin-60S ribosomal protein L40	UBA52	1.5	0.01
O75635-2	Serpin B7	SERPINB7	1.4	<0.01
P13796	Plastin-2	LCP1	1.4	0.06
Q9Y6R7	IgGFc-binding protein	FCGBP	1.4	0.38
Q13765	Nascent polypeptide-associated complex subunit alpha	NACA	1.4	0.21
Q99497	Protein deglycase DJ-1	PARK7	1.4	<0.01
Q9NP55-2	BPI fold-containing family A member 1	BPIFA1	1.4	0.55
P62158	Calmodulin	CALM1	1.4	0.07
P52565	Rho GDP-dissociation inhibitor 1	ARHGDIA	1.4	0.03
O75083	WD repeat-containing protein 1	WDR1	1.4	0.14
P04062-4	Glucosylceramidase	GBA	1.4	0.02
Q5VT79	Annexin A8-like protein 2	ANXA8L2	1.3	0.12
P60842	Eukaryotic initiation factor 4A-I	EIF4A1	1.3	0.50
Q9NQC3-2	Reticulon-4	RTN4	1.3	0.23
Q9NZT1	Calmodulin-like protein 5	CALML5	1.3	0.10
Q9Y2T3	Guanine deaminase	GDA	1.3	0.18
Q06AU7	Ras-related protein Rab-1B	RAB1B	1.3	0.10
P05090	Apolipoprotein D	APOD	1.3	0.36
P54920	Alpha-soluble NSF attachment protein	NAPA	1.3	0.05
Q9ULV0	Unconventional myosin-Vb	MYO5B	1.3	0.38
P07237	Protein disulfide-isomerase	P4HB	1.3	0.50
Q15517	Corneodesmosin	CDSN	1.2	0.07
P40926	Malate dehydrogenase, mitochondrial	MDH2	1.2	0.26
P20073-2	Annexin A7	ANXA7	1.2	0.44
P59998	Actin-related protein 2/3 complex subunit 4	ARPC4	1.2	0.55
P01833	Polymeric immunoglobulin receptor	PIGR	1.2	0.61
P60662-2	Myosin light polypeptide 6	MYL6	1.2	0.43
P56537	Eukaryotic translation initiation factor 6	EIF6	1.2	0.18
P06702	Protein S100-A9	S100A9	1.2	0.71
Q03591	Complement factor H-related protein 1	CFHR1	1.2	0.64
Q8TAA3-2	Proteasome subunit alpha type-7-like	PSMA8	1.2	0.07
Q5VVQ6-2	Ubiquitin thioesterase OTU1	YOD1	1.2	0.35
P04075	Fructose-bisphosphate aldolase A	ALDOA	1.2	0.54
P01024	Complement C3	C3	1.2	0.56
P25789	Proteasome subunit alpha type-4	PSMA4	1.2	0.08
P22392	Nucleoside diphosphate kinase B	NME2	1.2	0.58
P02787	Serotransferrin	TF	1.2	0.69
P05164-2	Myeloperoxidase	MPO	1.2	0.86
P55072	Transitional endoplasmic reticulum ATPase	VCP	1.1	0.71
P09972	Fructose-bisphosphate aldolase C	ALDOC	1.1	0.56
P02679-2	Fibrinogen gamma chain	FGG	1.1	0.74
A0A0A0MRZ7	Ig kappa chain V-II region RPMI 6410	IGKV2D-26	1.1	0.86
P19105	Myosin regulatory light chain 12A	MYL12A	1.1	0.49
Q02413	Desmoglein-1	DSG1	1.1	0.34
P10909-3	Clusterin	CLU	1.1	0.82
P04080	Cystatin-B	CSTB	1.1	0.61
Q0Z8U2	40S ribosomal protein S3	RPS3	1.1	0.74

P25787	Proteasome subunit alpha type-2	PSMA2	1.1	0.51
O75342	Arachidonate 12-lipoxygenase, 12R-type	ALOX12B	1.1	0.47
P49189	4-trimethylaminobutyraldehyde dehydrogenase	ALDH9A1	1.1	0.66
Q29545			1.1	NA
P50395	Rab GDP dissociation inhibitor beta	GDI2	1.0	0.85
P21753			1.0	NA
P02545-5	Prelamin-A/C	LMNA	1.0	0.90
Q710C4			1.0	0.73
O60784-3	Target of Myb protein 1	TOM1	1.0	0.87
P04792	Heat shock protein beta-1	HSPB1	1.0	0.95
P62495	Eukaryotic peptide chain release factor subunit 1	ETF1	1.0	0.96
Q08554-2	Desmocollin-1	DSC1	1.0	0.97
P21333-2	Filamin-A	FLNA	1.0	0.96
P60900	Proteasome subunit alpha type-6	PSMA6	1.0	0.82
P06454-2	Prothymosin alpha	PTMA	1.0	0.90
P68871	Hemoglobin subunit beta	HBB	1.0	0.88
P32119	Peroxiredoxin-2	PRDX2	1.0	0.74
P00558	Phosphoglycerate kinase 1	PGK1	1.0	0.83
P31151	Protein S100-A7	S100A7	0.9	0.85
P28066	Proteasome subunit alpha type-5	PSMA5	0.9	0.66
P13639	Elongation factor 2	EEF2	0.9	0.89
Q9TSX9			0.9	0.48
P63261	Actin, cytoplasmic 2	ACTG1	0.9	0.72
Q9HCY8	Protein S100-A14	S100A14	0.9	0.68
P22735	Protein-glutamine gamma-glutamyltransferase K	TGM1	0.9	0.54
P07737	Profilin-1	PFN1	0.9	0.79
Q4GWZ2	40S ribosomal protein SA	RPSA	0.9	0.74
Q01518	Adenylyl cyclase-associated protein 1	CAP1	0.9	0.65
Q64L94	Proteasome activator complex subunit 1	PSME1	0.9	0.65
P31949	Protein S100-A11	S100A11	0.9	0.63
O15144	Actin-related protein 2/3 complex subunit 2	ARPC2	0.9	0.63
P08835			0.9	0.89
P52907	F-actin-capping protein subunit alpha-1	CAPZA1	0.9	0.64
P10599	Thioredoxin	TXN	0.9	0.20
P98088	Mucin-5AC	MUC5AC	0.9	0.66
P28070	Proteasome subunit beta type-4	PSMB4	0.9	0.24
P26234-2	Vinculin	VCL	0.9	0.27
Q9UL46	Proteasome activator complex subunit 2	PSME2	0.8	0.01
P18510-4	Interleukin-1 receptor antagonist protein	IL1RN	0.8	0.39
Q01105-3	Protein SET	SET	0.8	0.42
P05386	60S acidic ribosomal protein P1	RPLP1	0.8	0.29
Q14574-2	Desmocollin-3	DSC3	0.8	0.09
P49419-4	Alpha-aminoadipic semialdehyde dehydrogenase	ALDH7A1	0.8	0.14
Q8IW75	Serpin A12	SERPINA12	0.8	0.32
P22528	Cornifin-B	SPRR1B	0.8	0.74
P02808	Statherin	STATH	0.8	0.80
P47929	Galectin-7	LGALS7	0.8	0.49
P00338	L-lactate dehydrogenase A chain	LDHA	0.8	0.48
P80229			0.8	0.23
Q92820	Gamma-glutamyl hydrolase	GGH	0.8	0.22
Q9UIV8	Serpin B13	SERPINB13	0.8	0.03
Q9Y3F4	Serine-threonine kinase receptor-associated protein	STRAP	0.8	0.04
P0C0L4-2	Complement C4-A	C4A	0.7	0.06
P31947	14-3-3 protein sigma	SFN	0.7	0.15
P81605	Dermcidin	DCD	0.7	0.28
Q9UGM3-9	Deleted in malignant brain tumors 1 protein	DMBT1	0.7	0.52
P30086	Phosphatidylethanolamine-binding protein 1	PEBP1	0.7	0.01
P34932	Heat shock 70 kDa protein 4	HSPA4	0.7	0.25

P05089	Arginase-1	ARG1	0.7	<0.01
P07384	Calpain-1 catalytic subunit	CAPN1	0.7	<0.01
P50452-2	Serpin B8	SERPINB8	0.7	0.12
Q9GZP4-2	PITH domain-containing protein 1	PITHD1	0.7	0.03
P36952	Serpin B5	SERPINB5	0.7	0.16
P07339	Cathepsin D	CTSD	0.7	0.01
P05387	60S acidic ribosomal protein P2	RPLP2	0.7	0.06
A8K2U0	Alpha-2-macroglobulin-like protein 1	A2ML1	0.7	0.24
Q9UI42	Carboxypeptidase A4	CPA4	0.6	0.01
Q6ZVX7	F-box only protein 50	NCCRP1	0.6	0.01
P20933	N(4)-(beta-N-acetylglucosaminyl)-L-asparaginase	AGA	0.6	0.29
Q5T749	Keratinocyte proline-rich protein	KPRP	0.6	0.02
P01040	Cystatin-A	CSTA	0.6	0.04
P27482	Calmodulin-like protein 3	CALML3	0.6	0.01
Q13867	Bleomycin hydrolase	BLMH	0.6	<0.01
P01876	Ig alpha-1 chain C region	IGHA1	0.6	0.10
P14923	Junction plakoglobin	JUP	0.6	0.02
Q01469	Fatty acid-binding protein, epidermal	FABP5	0.6	0.01
Q96G03	Phosphoglucomutase-2	PGM2	0.6	<0.01
P00491	Purine nucleoside phosphorylase	PNP	0.6	<0.01
Q8WVV4	Protein POF1B	POF1B	0.6	0.06
P02067			0.5	0.28
P32455	Interferon-induced guanylate-binding protein 1	GBP1	0.5	0.04
Q96P63	Serpin B12	SERPINB12	0.5	<0.01
P31025	Lipocalin-1	LCN1	0.5	0.05
P15924	Desmoplakin	DSP	0.5	0.01
P11708			0.5	0.13
P63167	Dynein light chain 1, cytoplasmic	DYNLL1	0.5	0.08
Q06210-2	Glutamine--fructose-6-phosphate aminotransferase [isomerizing] 1	GFPT1	0.5	0.55
P42357	Histidine ammonia-lyase	HAL	0.5	<0.01
P35908	Keratin, type II cytoskeletal 2 epidermal	KRT2	0.5	<0.01
P09228	Cystatin-SA	CST2	0.4	NA
O95969	Secretoglobin family 1D member 2	SCGB1D2	0.4	0.02
P31944	Caspase-14	CASP14	0.4	<0.01
P05109	Protein S100-A8	S100A8	0.4	0.03
P29508	Serpin B3	SERPINB3	0.4	<0.01
P06733	Alpha-enolase	ENO1	0.4	<0.01
O75223	Gamma-glutamylcyclotransferase	GGCT	0.4	<0.01
P60174-1	Triosephosphate isomerase	TPI1	0.4	<0.01
P14618	Pyruvate kinase PKM	PKM	0.4	0.03
P13473-2	Lysosome-associated membrane glycoprotein 2	LAMP2	0.4	0.02
P04406-2	Glyceraldehyde-3-phosphate dehydrogenase	GAPDH	0.4	<0.01
P02788	Lactotransferrin	LTF	0.4	0.07
P25311	Zinc-alpha-2-glycoprotein	AZGP1	0.4	<0.01
P19971	Thymidine phosphorylase	TYMP	0.3	0.03
P01037	Cystatin-SN	CST1	0.3	0.01
Q9HC84	Mucin-5B	MUC5B	0.3	0.02
P81245			0.3	0.01
A0A0C4DH55	Ig kappa chain V-III region POM	IGKV3D-7	0.2	<0.01
P04745	Alpha-amylase 1	AMY1A	0.2	<0.01
Q08188	Protein-glutamine gamma-glutamyltransferase E	TGM3	0.2	<0.01
P01036	Cystatin-S	CST4	0.2	<0.01
P12273	Prolactin-inducible protein	PIP	0.2	<0.01
P61626	Lysozyme C	LYZ	0.2	<0.01
Q53RT3	Retroviral-like aspartic protease 1	ASPRV1	0.1	<0.01
P14735	Insulin-degrading enzyme	IDE	0.1	<0.01
Q96DA0	Zymogen granule protein 16 homolog B	ZG16B	0.0	<0.01

Table S4. Proteins present at different amounts Early Post C compared with Pre C				
**Majority protein IDs**	**Protein names**	**Gene names**	**Fold change Post C/Pre C**	**p-value**
O02705	Heat shock protein HSP 90-alpha	HSP90AA1	14.9	0.03
P00338	L-lactate dehydrogenase A chain	LDHA	9.7	0.06
Q2XVP4	Tubulin alpha-1B chain	TUBA1B	9.1	0.04
Q16777	Histone H2A type 2-C	HIST2H2AC	8.3	0.06
Q5VTE0	Putative elongation factor 1-alpha-like 3	EEF1A1P5	7.0	0.08
A8K2U0	Alpha-2-macroglobulin-like protein 1	A2ML1	6.6	0.02
P04075	Fructose-bisphosphate aldolase A	ALDOA	4.8	0.07
P01024	Complement C3	C3	4.0	0.08
P62937	Peptidyl-prolyl cis-trans isomerase A	PPIA	3.4	0.03
P63261	Actin, cytoplasmic 2	ACTG1	3.3	0.07
P63104	14-3-3 protein zeta/delta	YWHAZ	2.4	0.02
Q9BWD1	Acetyl-CoA acetyltransferase, cytosolic	ACAT2	0.7	0.10
P05089	Arginase-1	ARG1	0.4	0.07
O75223	Gamma-glutamylcyclotransferase	GGCT	0.4	0.09
O95336	6-phosphogluconolactonase	PGLS	0.4	0.02
O95969	Secretoglobin family 1D member 2	SCGB1D2	0.3	0.08

Table S5. Proteins present at different amounts Late Post C compared with Pre C				
**Majority protein IDs**	**Protein names**	**Gene names**	**Fold change Late Post C/Pre C**	**p-value**
P19971	Thymidine phosphorylase	TYMP	12.3	0.08
P14618	Pyruvate kinase PKM	PKM	10.1	0.10
Q16777	Histone H2A type 2-C	HIST2H2AC	9.2	0.05
O02705	Heat shock protein HSP 90-alpha	HSP90AA1	7.4	0.05
A8K2U0	Alpha-2-macroglobulin-like protein 1	A2ML1	6.5	0.08
P09211	Glutathione S-transferase P	GSTP1	6.0	0.03
P11142	Heat shock cognate 71 kDa protein	HSPA8	4.5	0.03
Q4GWZ2	40S ribosomal protein SA	RPSA	4.3	0.06
P31947	14-3-3 protein sigma	SFN	3.8	0.05
P06733	Alpha-enolase	ENO1	3.4	0.02
P47929	Galectin-7	LGALS7	2.8	0.05
P63261	Actin, cytoplasmic 2	ACTG1	2.5	0.08
P06748	Nucleophosmin	NPM1	0.6	<0.01
Q08554	Desmocollin-1	DSC1	0.6	0.06
P10809	60 kDa heat shock protein, mitochondrial	HSPD1	0.3	0.01
P01624	Ig kappa chain V-III region POM	IGKV3D-7	0.2	0.05
P61626	Lysozyme C	LYZ	0.1	0.07

Table S6: Proteins present at different amounts Late Post PE compared with Pre PE in the animals treated with placebo (n=6)

Majority protein IDs	Protein names	Gene names	Fold change Late Post PE/Pre, placebo animals	p-value
P61626	Lysozyme C	LYZ	3.2	0.09
P14174	Macrophage migration inhibitory factor	MIF	3.1	0.05
Q52NJ1	Ras-related protein Rab-11A	RAB11A	2.4	0.07
Q6ZVX7	F-box only protein 50	NCCRP1	2.3	0.01
P60900	Proteasome subunit alpha type-6	PSMA6	1.6	<0.01
P28066	Proteasome subunit alpha type-5	PSMA5	1.3	0.06
P06396	Gelsolin	GSN	1.2	0.06
P30086	Phosphatidylethanolamine-binding protein 1	PEBP1	0.6	0.08
P16989	Y-box-binding protein 3	YBX3	0.4	0.03
P01619	Ig kappa chain V-III region B6	IGKV3D-20	0.4	0.07
P07108	Acyl-CoA-binding protein	DBI	0.3	0.05
P80188	Neutrophil gelatinase-associated lipocalin	LCN2	0.2	<0.01

Table S7. Proteins for prediction of Early Post PE versus Early Post C.

Major protein ID	Gene name	Coefficient*
P63261	ACTG1	-0.0286495
Q2XVP4	TUBA1B	-0.5719353
Q02413	DSG1	0.0736192

* Positive values mean that an increase in expression increases the probability of PE, whereas a negative value means that an increase in expression decreases the probability of PE

Table S8. Proteins for prediction of Early Post PE versus Pre PE.

Major protein ID	Gene name	Coefficient*
A8K2U0	A2ML1	-0.0073061
O02705	HSP90AA1	-0.0110015
O75223	GGCT	-0.0112219
O75635-2	SERPINB7	-0.0477659
P00338	LDHA	0.0188611
P00558	PGK1	-0.0284317
P01024	C3	-0.0352144
P01040	CSTA	0.0062836
P01876	IGHA1	0.0162831
P01965	HBA	-0.0066456
P04792	HSPB1	-0.0434449
P05089	ARG1	-0.0184193
P05109	S100A8	-0.0114851
P05387	RPLP2	-0.0213611
P06753-5	TPM3	0.0334131
P07339	CTSD	-0.0419630
P08835	ALB	0.0093072
Q4GWZ2	RPSA	-0.0136737
P09211	GSTP1	-0.0163435
P09571	TF	0.0055013
P63053	UBA52	0.0396376
P10599	TXN	-0.0370181
P10668	CFL1	-0.0066365
P12273	PIP	0.0104544
P13639	EEF2	-0.0232908
P15924	DSP	-0.0174612
P16401	H1-5	-0.0126885
P19971	TYMP	-0.0218455
P22735	TGM1	-0.0687911
P25311	AZGP1	0.0372070
P27482	CALML3	-0.0157583
P29508	SERPINB3	-0.0166842
P31025	LCN1	0.0137401
P35579	MYH9	-0.0407100
P42357	HAL	0.0318255
P47929	LGALS7	-0.0028848
P48594	SERPINB4	-0.0369825
P55072	VCP	-0.0225843
P56537	EIF6	-0.0456300

P58107	EPPK1	-0.0005712
P60174-1	TPI1	-0.0385885
P62158	CALM1	-0.0003029
Q5VTE0	EEF1A1P5	-0.0202294
Q2XVP4	TUBA1B	-0.0106841
P80188	LCN2	-0.0520429
Q01469	FABP5	-0.0112143
Q06830	PRDX1	-0.0687322
Q13835-2	PKP1	-0.0262028
Q13867	BLMH	0.0385349
Q15517	CDSN	0.0911075
Q16777	H2AC20	-0.0121097
Q6ZVX7	NCCRP1	0.0061640
Q96P63	SERPINB12	0.0359381
Q99497	PARK7	-0.0259430
Q9NZH8	IL36G	-0.0237314
Q9NZT1	CALML5	-0.0576804
Q9UI42	CPA4	0.0633854
* Positive values mean that an increase in expression increases the probability of PE, whereas a negative value means that an increase in expression decreases the probability of PE		

Table S9. Proteins for prediction of Late Post PE versus Pre PE.		
Major Protein ID´s	Gene name	Coefficient*
P01876	IGHA1	0.0986191
P05387	RPLP2	-0.3084017
P08835	ALB	0.1298375
P80188	LCN2	-0.2891508
Q9NZH8	IL36G	-0.1614609
Positive values mean that an increase in expression increases the probability of PE, whereas a negative value means that an increase in expression decreases the probability of PE.		
